# Evaluation of Decreased Kidney Function in Dogs Receiving Carboplatin: A Retrospective Cohort Study of 98 Dogs (2006–2024)

**DOI:** 10.1111/vco.13069

**Published:** 2025-06-05

**Authors:** Andrew D. Yale, Alvina So, Alexandra Guillén, Isabelle Desmas‐Bazelle, Francesco Rogato, Rosanne E. Jepson

**Affiliations:** ^1^ Department of Clinical Science and Services Royal Veterinary College Hertfordshire UK; ^2^ Washington State University Pullman WA USA

**Keywords:** canine, chemotherapy, oncology, renal, toxicity

## Abstract

Decreased kidney function is observed in some people receiving carboplatin, but limited literature explores this in dogs. The aim of this study was to evaluate the incidence and risk factors for decreased kidney function in dogs receiving carboplatin. A single‐institute retrospective cohort study compared the incidence of decreased kidney function between non‐azotaemic dogs receiving carboplatin and an age‐ and weight‐matched cancer‐bearing control group not receiving chemotherapy. Change in creatinine concentration and a linear mixed effects model were used to compare trends in creatinine between groups. Decreased kidney function was defined as a sustained increase in creatinine ≥ 26.5 μmol/L on ≥ 2 consecutive measurements compared to baseline; the VCOG‐CTCAE v2 grading system for increased creatinine was also applied. Risk factors were explored. Ninety‐eight dogs were included (*n* = 49/group). There was no difference in median change in creatinine concentration (+2.0 μmol/L; *p* = 0.311) or creatinine trends (*p* = 0.958) across the study period between groups. Incidence of decreased kidney function was low and did not significantly differ between groups (carboplatin group *n* = 4 [8.2%]; control group *n* = 2 [4.1%]; *p* = 0.678); no risk factors were identified. There was no difference in the frequency of VCOG grade one (*p* = 0.731), two (*p* = 0.641) or three (*p* = 0.429) creatinine adverse events between groups. Non‐azotaemic dogs receiving carboplatin do not have a significantly increased short‐term risk of decreased kidney function compared to those not receiving chemotherapy, although the numerical increase in incidence in dogs receiving carboplatin could be clinically relevant. Larger studies should aim to explore this further and investigate carboplatin's impact on subclinical and long‐term renal function.

## 1 Introduction

Platinum agents, including cisplatin and carboplatin, primarily act by covalently binding to DNA and forming crosslinks, which inhibit DNA replication, leading to cell cycle arrest and apoptosis [1]. Platinum is the core and common element of both agents, with the main molecular difference being the reactive leaving groups (chloride and cyclobutane‐decarboxylate for cisplatin and carboplatin, respectively) [2]. These have significant pharmacokinetic effects and determine differences in intratumoural drug concentrations achieved by each agent [3]. In people, cisplatin and carboplatin are effective against similar tumour types although cisplatin is considered more potent and toxic [1]. Adverse effects (AEs) related to cisplatin administration in people include gastrointestinal toxicity, nephrotoxicity, neurotoxicity, and ototoxicity, whereas carboplatin AEs primarily include myelosuppression with a lower risk of renal or neurological toxicity [4].

There are multiple mechanisms of cisplatin nephrotoxicity including DNA damage, mitochondrial dysfunction, oxidative stress, inhibition of protein synthesis, and induction of renal tubular epithelial cell apoptosis [5, 6, 7, 8]. Cisplatin accumulates intracellularly following uptake through the organic cation transporter OCT2 on renal tubular epithelial cells and can reach approximately five times the serum concentration [9, 10]. Carboplatin does not interact with OCT2, contributing to its reduced nephrotoxicity [11, 12]. Clinically, cisplatin‐induced nephrotoxicity typically manifests as acute tubular injury with a higher incidence and severity of renal dysfunction, whereas carboplatin is associated with a significantly lower risk and often presents as a subclinical reduction of kidney function [13]. Carboplatin nephrotoxicity is particularly seen in patients with pre‐existing impaired renal function or in those receiving multiple courses of treatment and reaching high cumulative doses [13, 14, 15, 16].

There is limited literature evaluating the risk of nephrotoxicity in dogs receiving carboplatin. Two phase I/toxicology studies from 1987 and 1993 did not identify azotaemia in three and 28 dogs, respectively, although transient mild proteinuria was documented in the earlier study [17, 18]. One study describing chemotherapy overdoses included one case of carboplatin overdose (377 mg/m^2^) in a dog; no nephrotoxicity was reported, but it is unclear if this was specifically evaluated [19]. A prospective study assessing the safety of intravenous versus subcutaneous carboplatin in five dogs did not report nephrotoxicity [20]. Other studies involving carboplatin administration in dogs are primarily retrospective and focused on clinical outcomes in various tumour types; where toxicity was evaluated, there was no consistent or specific monitoring for nephrotoxicity [21, 22, 23, 24, 25, 26]. From the aforementioned studies, only one dog had a reported increase in creatinine following carboplatin administration, although the cause was unknown, and it was not possible to link directly to carboplatin administration [26].

Due to the lack of literature specifically assessing the risk of decreased kidney function in dogs, the aim of this study was to evaluate the incidence and risk factors for developing decreased kidney function in dogs receiving carboplatin.

## 2 Materials and Methods

### 2.1 Case Selection

Medical records from the Royal Veterinary College's Queen Mother Hospital for Animals (United Kingdom) between 2006 and 2024 were reviewed for this retrospective cohort study.

**FIGURE 1:**
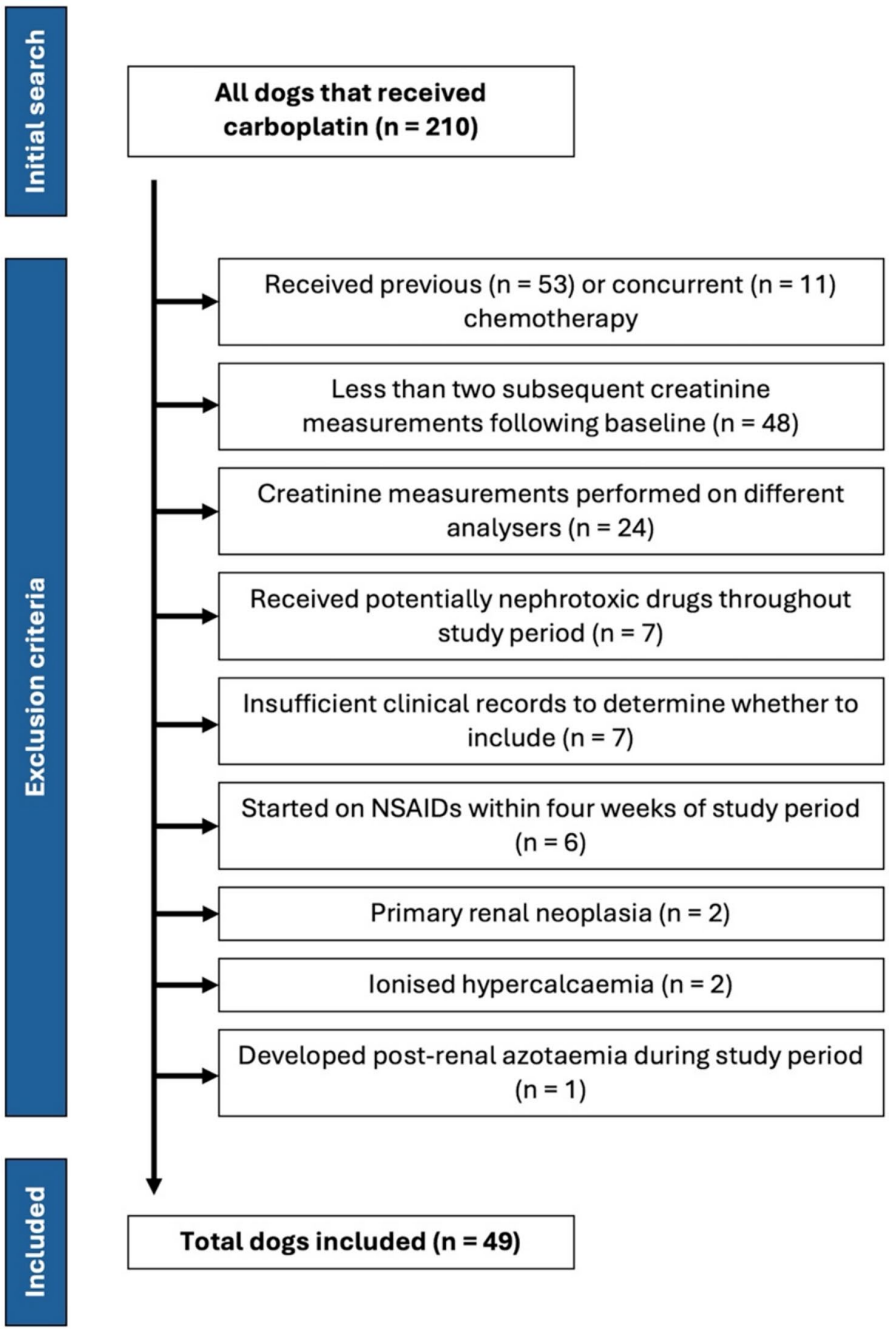
Flow diagram depicting the identification, inclusion and exclusion of dogs receiving carboplatin; the institute's clinical records database was searched for all dogs that received one or more doses of carboplatin. *NSAIDs = non‐steroidal anti‐inflammatory drugs*.

Dogs were included in the carboplatin group if they had malignant neoplasia and received ≥ 1 dose of intravenous carboplatin as part of a single‐agent chemotherapy protocol (Figure 1). An age‐ and weight‐matched control group was also created, consisting of dogs diagnosed with malignant neoplasia that were not receiving cytotoxic chemotherapy or molecular targeted therapies during or prior to the study period. For the control group, medical records were searched for dogs with cancer types that are typically managed surgically with subsequent monitoring (e.g., apocrine gland anal sac adenocarcinoma [AGASACA], pulmonary carcinoma, hepatocellular carcinoma [HCC]). Longlisted cases were reviewed randomly until the same number of dogs was included compared to the carboplatin group. The mean age and median weight automatically matched the carboplatin group without requiring further exclusion and inclusion of cases.

All dogs were required to have a baseline creatinine concentration within the reference interval on the machine where their sample was analysed, and at least two subsequent creatinine measurements. For dogs that received carboplatin, the baseline creatinine was required to be within three months preceding the first carboplatin dose. In both the carboplatin and control groups, creatinine measurements for individual dogs were required to be performed on the same analyser, although analysers could differ between dogs.

Dogs were excluded if they had primary or secondary renal neoplasia, ionised hypercalcaemia, developed azotaemia due to a documented post‐renal cause, if serum creatinine concentration increased by ≥ 26.5 μmol/L from baseline before administration of carboplatin, or if dogs were receiving fluid therapy at the time of any creatinine measurement. They were also excluded if there was use of a drug considered potentially nephrotoxic (e.g., bisphosphonates) during the study period. Non‐steroidal anti‐inflammatory drugs (NSAIDs) were permitted in both groups if these had been administered for a minimum of four weeks prior to study inclusion and records showed creatinine concentration within normal limits prior to starting NSAIDs and at the baseline measurement at the start of the study period.

### 2.2 Clinical Data

Clinical information compiled included age, sex, neuter status, breed, body weight, and diagnosis. For the carboplatin group, the date and dose (mg/m^2^) of carboplatin treatments were recorded. For both groups, baseline creatinine was recorded in addition to the dates and creatinine concentrations for all available follow‐up measurements. Additional information collected for both groups, where available, included: diagnostic imaging data throughout the study period, baseline and subsequent urinalysis, blood pressure and fundic examination data, and the number of sedations, general anaesthetics (GA) and contrast (iohexol) computed tomography (CT) performed.

The study period was defined as the time between the baseline and final creatinine measurement for each dog. Creatinine measurements were no longer recorded once a dog started a different chemotherapy, targeted therapy, nephrotoxic drug, or other exclusion criteria were met (e.g., creatinine recorded on a different analyser). No minimum follow‐up period was required.

### 2.3 Decreased Kidney Function

Decreased kidney function was defined as a sustained increase in creatinine of ≥ 26.5 μmol/L compared to baseline on ≥ 2 consecutive measurements. This was based on the Veterinary Cooperative Oncology Group Common Terminology Criteria for Adverse Events (VCOG‐CTCAE) v2 acute kidney injury (AKI) grading system, which is modified from the International Renal Interest Society (IRIS) AKI grading [27, 28]. However, in the current study, a sustained increase in creatinine was required to exclude dogs with transient increases in creatinine (e.g., pre‐renal causes). Creatinine values were also categorised according to the VCOG‐CTCAE v2 grading system for increased creatinine compared to baseline (Table 1).

**TABLE 1:** Grading systems used for evaluation of decreased kidney function in dogs receiving carboplatin chemotherapy, from Veterinary Cooperative Oncology Group Common Terminology Criteria for Adverse Events (VCOG‐CTCAE) v2 [27]. Semi‐colon indicates “or”.

Adverse Event	Grade (VCOG‐CTCAE v2)				
	1	2	3	4	5
**Acute kidney injury (modified IRIS grade)**	Creatinine ≤ 1.6 mg/dL (140 μmol/L) with increase of ≥ 0.3 mg/dL (26.5 μmol/L) from baseline	Creatinine 1.7–2.5 mg/dL (141—220 μmol/L); fluid responsive oliguria/anuria within 48 h	Creatinine 2.6–5.0 mg/dL (221—439 μmol/L); fluid therapy (IV or SC) and/or renal diet indicated	Creatinine > 5.1 mg/dL (440 μmol/L); renal replacement therapy (RRT) indicated	Death
**Creatinine, high**	> 1.0–1.5x baseline; > ULN—1.5x ULN	> 1.5–3.0x baseline; > 1.5–2.0x ULN	> 3x baseline; > 2.0–3.0x ULN	> 3.0x ULN	—

Abbreviations: IRIS, International Renal Interest Society; ULN, upper limit of normal.

### 2.4 Statistical Analysis

Frequency and proportion were used to report categorical variables. The Shapiro–Wilk test was used to assess the normality of continuous data, which was reported as the mean and standard deviation (SD) for normally distributed data, and the median and range for non‐normally distributed data. The unpaired t‐test and Mann–Whitney U test were used to compare normally and non‐normally distributed continuous data, respectively, between the carboplatin and control groups. Fisher's exact and chi‐squared tests were used to assess associations between sets of categorical variables, and binary logistic regression was used to compare continuous variables against the development of decreased kidney function; results were presented as relative risk (RR) or odds ratio (OR) and 95% confidence intervals (CI). The linear mixed effects model was used to compare creatinine concentrations and trends over time between groups; group, time, and their interaction were included in the model as fixed effects, and the dog identification number was included as a random effect. The normality of the residuals was assessed visually. *P*‐values ≤ 0.05 were considered significant.

### 2.5 Ethics Statement

As this study was retrospective and anonymised, full ethical approval was not required; this was confirmed via the institution's Social Sciences Research Ethical Review Board (URN SR2024‐0133R0808).

### 2.6 Cell Line Authentication Statement

No cell lines were used in this study.

## 3 Results

### 3.1 Group Characteristics and Comparison

Forty‐nine dogs were included in the carboplatin group. The mean age was 9.2 years (SD = 2.1), and median weight was 24.5 kg (range, 5.7–49.4). Two (4.1%) dogs were male entire (ME), 22 (44.9%) male neutered (MN), and 25 (51%) female neutered (FN). The most common breeds represented were crossbreed (*n* = 12; 24.5%), Labrador retriever (*n* = 5; 10.2%) and cocker spaniel (*n* = 4; 8.2%). The most common cancer types included appendicular osteosarcoma (*n* = 21; 42.9%), AGASACA (*n* = 9; 18.4%) and pulmonary adenocarcinoma (*n* = 4; 8.2%). Thirty‐six (73.5%) dogs had surgery prior to the study period, and 13 (26.5%) had no prior treatment. One (2.0%) dog had a course of hyperfractionated radiation therapy (RT) during the study period. Forty‐nine dogs were also included in the control group. The mean age was 9.7 years (SD = 2.1), and the median weight was 16.8 kg (range, 6.9–50.0). Four (8.2%) dogs were ME, 27 (55.1%) were MN, and 18 (36.7%) were FN. The most common breeds represented were crossbreed (*n* = 9; 18.4%), cocker spaniel (*n* = 9; 18.4%) and Labrador retriever (*n* = 5; 10.2%). The most common cancer types included AGASACA (*n* = 20; 40.8%), pulmonary adenocarcinoma (*n* = 8; 16.3%) and HCC (*n* = 5; 10.2%). Complete data regarding the breed and cancer type distributions for all dogs are provided in the Supplementary Information. Forty‐five (91.8%) dogs had surgery prior to the study period, and four (8.2%) dogs had no treatment. Three (6.1%) dogs underwent hyperfractionated RT during the study period.

In the carboplatin group, thirty‐two (65.3%) dogs had baseline abdominal imaging (CT = 27, ultrasound = 5) and sixteen (32.7%) had baseline urinalysis (Table 2). One (2.0%) dog had urine protein: creatinine ratio (UPCR) measured, which was mildly increased (0.79; reference interval 0.0–0.5). One (2.0%) dog had baseline non‐invasive blood pressure (NIBP) measured, and was normal ( < 140 mmHg); the same dog was the only dog to have had a baseline fundic examination, which revealed unilateral mild partial retinal detachment, of which the cause was not identified. In the control group, forty‐four dogs (89.8%) had baseline abdominal imaging (CT = 38, ultrasound = 6) and seven (14.3%) had baseline urinalysis (Table 2). None had baseline UPCR, blood pressure measurement, or fundic examination.

**TABLE 2:** Frequency of abdominal imaging and urinalysis abnormalities, in addition to risk factor exposure, in the carboplatin and control groups.

		Carboplatin	Control
**Renal changes on baseline abdominal imaging (n [%])**	Infarcts	7 (21.9%)	2 (4.5%)
	Cysts	2 (6.3%)	5 (11.4%)
	Mild bilateral renal pelvis dilation	1 (3.1%)	—
	Mild bilateral renal pelvis mineralisation	1 (3.1%)	—
	Single small nephrolith	1 (3.1%)	—
	Small bilateral nephroliths	—	1 (2.3%)
**Urinalysis (n [%], or median [range])**	USG (median, range)	1.028 (1.010–1.050)	1.027 (1.010 – > 1.050)
	pH (median, range)	6.5 (6.0–9.0)	6.0 (5.5–9.0)
	Protein (trace)	4 (25%)	1 (14.3%)
	Protein (1+)	6 (37.5%)	1 (14.3%)
	Protein (2+)	1 (6.3%)	1 (14.3%)
	Bilirubin (1+)	5 (31.3%)	1 (14.3%)
	Bilirubin (2+)	1 (6.3%)	—
	Ketones (trace)	1 (6.3%)	2 (28.6%)
	Blood (4+)	1 (6.3%)	—
**Risk factors for decreased kidney function (n [%], or median [range])**	Contrast CT scans (median, range)	1 (0–3)	1 (0–3)
	Sedation/GA events (median, range)	1 (0–22)	2 (0–20)
	COX‐2 NSAIDs	20 (40.8%)	15 (30.6%)

Abbreviations: CT, computed tomography; GA, general anaesthesia; NSAIDs, non‐steroidal anti‐inflammatory drugs; USG, urine specific gravity.

The number of contrast CT scans, sedation and GA events, and dogs receiving NSAIDs in each group is also presented in Table 2.

In the carboplatin group, there was a total of 238 carboplatin administrations during the study period. The median number of carboplatin doses per dog was five (range, 1–12), with a median interval between treatments of 22 days (range, 20–71). The median dose per carboplatin administration was 300 mg/m^2^ (range, 180–320), and the median cumulative carboplatin dose per dog was 1330 mg/m^2^ (range, 300–3100).

The median duration of the study period was 138 days (range, 21–598) and 197 days (range, 48–725) for the carboplatin and control groups, respectively.

There was no significant difference when comparing age (*p* = 0.247), bodyweight (*p* = 0.152), number of dogs with renal changes on baseline imaging (*p* = 0.161), number of dogs receiving NSAIDs (*p* = 0.399), number of contrast CT scans (*p* = 0.753) or number of sedation and GA events (*p* = 0.129) during the study period between groups. There was a significant difference in the duration of the study period between groups (*p* = 0.017).

### 3.2 Creatinine

In the carboplatin group, 198 creatinine measurements were recorded. The median number of creatinine measurements per dog was four (range, 3–7), and the median interval between measurements was 42 days (range, 7–455). The median creatinine concentration at baseline was 73.0 μmol/L (range, 31.0–148.0). The median creatinine concentration at last follow‐up was 76.0 μmol/L (range, 44.0–139.0).

In the control group, 149 creatinine measurements were recorded. The median number of creatinine measurements per dog was three (range, 3–4), and the median interval between measurements was 105 days (range, 5–524). The median creatinine concentration at baseline was 66.0 μmol/L (range, 31.0–124.0). The median creatinine concentration at last follow‐up was 73.0 μmol/L (range, 32.0–109.0).

**FIGURE 2:**
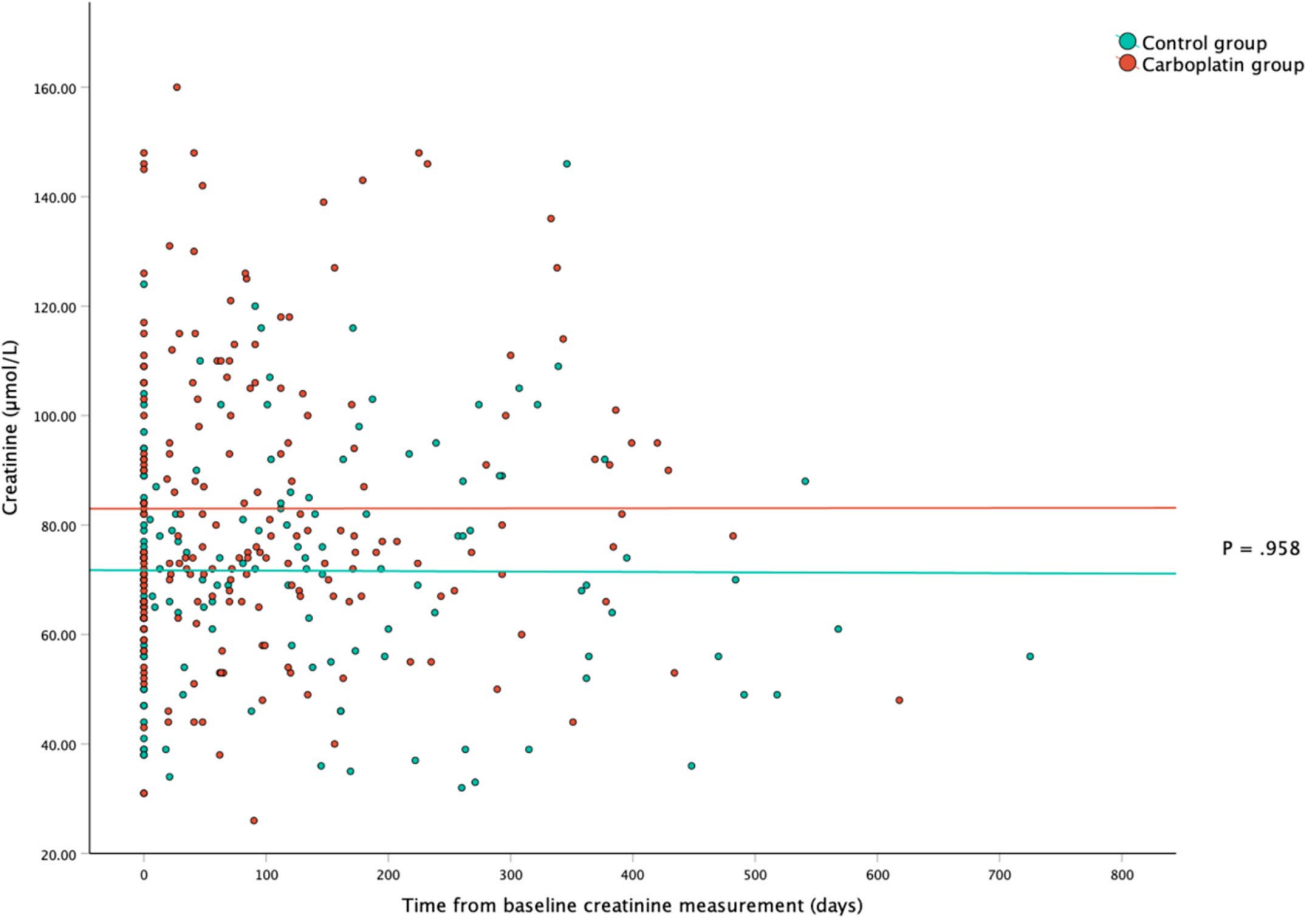
All creatinine values across the study period from the time of baseline measurement in dogs receiving carboplatin chemotherapy (red) and the control group of dogs not receiving carboplatin (green); lines represent average creatinine trends for each group. *P*‐value based on linear mixed effects modelling.

There was no significant difference in median change in creatinine concentration over the study period between groups (carboplatin group: +2.0 μmol/L [range, −34.0 – +61.0]; control group: +2.0 μmol/L [range, −35.0 – +44.0]; *p* = 0.311). Creatinine regression lines were also not significantly different between groups (*p* = 0.958; Figure 2).

### 3.3 Decreased Kidney Function

Decreased kidney function was observed in four (8.2%) dogs in the carboplatin group and two (4.1%) dogs in the control group. No dogs had more than one episode of decreased kidney function. There was no significant difference in the incidence of decreased kidney function between the carboplatin and control groups (*p* = 0.678; RR 2.00 [95% CI 0.38–10.42]). Pertinent clinicopathologic information from the dogs that experienced decreased kidney function in both groups is presented in Table 3.

**TABLE 3:** Clinicopathologic data from the six dogs that demonstrated decreased kidney function during the study period. † = bilateral renal infarcts.

Carboplatin Group										
	Signalment; Diagnosis	Time of first creatinine measurement ≥ 26.5 μmol/L from baseline, since starting carboplatin (days)	Cumulative carboplatin dose at point decreased kidney function was identified (mg/m^2^)	Maximum creatinine value recorded (μmol/L)	Maximum change in creatinine from baseline (μmol/L)	NSAIDs during study period	Renal changes on baseline imaging	Number of contrast CT scans during study period	Number of sedation and general anaesthetic events during study period	Renal imaging findings at time of/after documentation of decreased kidney function.
**Dog 1**	3.5y MN Labrador retriever; axial (rib) osteosarcoma	91	1200	95	+41	Yes	No	2	2	CT scan 62 days following documentation of decreased kidney function. No renal abnormalities reported.
**Dog 2**	11.9y FN Labrador retriever; pulmonary carcinoma	45	270	127	+61	No	Yes^†^	2	2	CT scan at time of documentation of decreased kidney function. Stable bilateral renal infarcts. No other renal abnormalities reported.
**Dog 3**	9.5y MN crossbreed; appendicular osteosarcoma	189	2400	148	+39	No	No	3	3	CT scan 225 days following documentation of decreased kidney function. No renal abnormalities reported.
**Dog 4**	7.6y FN greyhound; appendicular osteosarcoma	21	300	160	+60	No	No	1	2	Not performed.

Control Group										
	Signalment; Diagnosis	Time of first creatinine measurement ≥ 26.5 μmol/L from baseline, since start of study period (days)	‐	Maximum creatinine value recorded (μmol/L)	Maximum change in creatinine from baseline (μmol/L)	NSAIDs during study period	Renal changes on baseline imaging	Number of contrast CT scans during study period	Number of sedation and general anaesthetic events during study period	Renal imaging findings at time of/after documentation of decreased kidney function.
**Dog 1**	11.3y MN Japanese Akita Inu; thymoma	171	—	116	+51	No	Yes^†^	1	2	CT scan 168 days following documentation of decreased kidney function. Stable bilateral renal infarcts. No other renal abnormalities reported.
**Dog 2**	12.0y MN golden retriever; oral fibrosarcoma	346	—	146	+99	Yes	No	0	0	Not performed.

Abbreviations: CI, confidence interval; CT, computed tomography; GA, general anaesthesia; NSAID, non‐steroidal anti‐inflammatory drug; OR, odds ratio; SD, standard deviation.

In the carboplatin group, no risk factors for decreased kidney function were identified (Table 4). The median cumulative carboplatin dose received was marginally higher in dogs who demonstrated decreased kidney function, but this was not statistically significant. Variables were not analysed in a multivariable model as too few approached significance (*p* ≤ 0.10) to be included. Risk factors were not investigated in the control group due to the very small number of dogs meeting the definition for decreased kidney function.

**TABLE 4:** Univariate risk factor analysis for decreased kidney function in dogs receiving carboplatin.

Carboplatin Group				
Risk factor		Outcome	*P*‐value	OR (95% CI)
Age in months (mean, SD)	111.8 (3.6)	No decreased kidney function (*n* = 45)	0.288	0.98 (0.94–1.02)
	97.5 (21.3)	Decreased kidney function (*n* = 4)		
Bodyweight in kg (median, range)	23.0 (5.7–49.4)	No decreased kidney function (*n* = 45)	0.055	1.11 (1.00–1.24)
	39.8 (24.5–41.8)	Decreased kidney function (*n* = 4)		
Baseline creatinine in μmol/L (median, range)	73.0 (31.0–148.0)	No decreased kidney function (*n* = 45)	0.968	1.00 (0.96–1.04)
	83.0 (54.0–109.0)	Decreased kidney function (*n* = 4)		
Renal changes present on baseline imaging (n, %)	9 (31.0%)	No decreased kidney function (*n* = 29)	1.00	2.22 (0.13–39.64)
	1 (33.3%)	Decreased kidney function (*n* = 3)		
Number of carboplatin doses received (median, range)	5.0 (1.0–12.0)	No decreased kidney function (*n* = 45)	0.352	1.26 (0.78–2.03)
	5.50 (4.0–8.0)	Decreased kidney function (*n* = 4)		
Cumulative carboplatin dose in mg/m^2^ (median, range)	1260 (300–3100)	No decreased kidney function (*n* = 45)	0.216	1.00 (1.00–1.00)
	1535 (1208–2400)	Decreased kidney function (*n* = 4)		
Receiving NSAIDs (n, %)	19 (42.2%)	No decreased kidney function (*n* = 45)	0.636	0.46 (0.04–4.73)
	1 (25.0%)	Decreased kidney function (*n* = 4)		
Number of contrast CT scans (median, range)	1.0 (0.0–3.0)	No decreased kidney function (*n* = 45)	0.055	3.64 (0.97–13.64)
	2.0 (1.0–3.0)	Decreased kidney function (*n* = 4)		
Number of sedation/GA events (median, range)	1.0 (0.0–22.0)	No decreased kidney function (*n* = 45)	0.754	1.04 (0.81–1.34)
	2.0 (2.0–3.0)	Decreased kidney function (*n* = 4)		

Abbreviations: CI, confidence interval; CT, computed tomography; GA, general anaesthesia; NSAID, non‐steroidal anti‐inflammatory drug; OR, odds ratio; SD, standard deviation.

When utilising the VCOG‐CTCAE v2 grading system for increased creatinine, 34 (69.4%) dogs in the carboplatin group had at least one creatinine measurement classed as a grade one or higher AE. There was a total of 68 (34.3%) and 12 (6.1%) measurements categorised as a grade one or two increase in creatinine, respectively. In the control group, 36 (73.5%) dogs had at least one creatinine measurement classed as an AE. There was a total of 48 (32.2%), seven (4.7%), and one (0.7%) measurement categorised as a grade one, two, or three increase in creatinine, respectively. There was no significant difference between the frequency of VCOG grade one (*p* = 0.731; RR 1.07 [95% CI 0.79–1.44]), two (*p* = 0.641; RR 1.29 [95% CI 0.52–3.20]) or three (*p* = 0.429; RR 0.25 [95% CI 0.01–6.12]) creatinine AEs between the carboplatin and control groups.

## 4 Discussion

This retrospective study evaluated the incidence and risk factors for the development of decreased kidney function in non‐azotaemic dogs receiving carboplatin, compared to a matched control group of non‐azotaemic cancer‐bearing dogs not receiving chemotherapy. Overall, the findings indicate a low incidence of short‐term decreased kidney function, with no significant difference in frequency between groups. This larger study is in line with previous veterinary literature, although studies specifically addressing this topic are limited, and due to the relatively small study population, the increased incidence of decreased kidney function in the carboplatin group may be clinically relevant despite not being statistically significant [17, 18, 19, 20, 21, 22, 23, 24, 25, 26].

To increase the reliability of the results, dogs were only included in the carboplatin group if they had not received other chemotherapeutic or molecular targeted therapies prior to or during the study period as, occasionally, nephrotoxicity is reported with other agents such as cyclophosphamide and toceranib phosphate [29, 30]. Given the correlation between serum creatinine and lean body mass, and the potential for a decline in kidney function with age, the control group was matched for weight and age [31, 32, 33]. Inter‐analyser variability for serum creatinine concentrations can be high, so dogs were only included if all creatinine measurements throughout the study period were performed using the same analyser [34]. Dogs exposed to certain risk factors, such as sedation, GA, NSAID administration, and iohexol exposure, were permitted for study inclusion as these are frequently encountered in populations of small animal cancer patients and ensure the study results are applicable to the real‐world clinical setting. There was no significant difference in exposure to these risk factors between the carboplatin and control groups, so they were considered unlikely to influence the incidence of decreased kidney function between groups. In humans, NSAID nephrotoxicity is considered most likely to manifest during the first four weeks of administration [35]. Whilst NSAID nephrotoxicity has been reported in the veterinary literature, a RR period has not been identified. Given the high frequency of NSAID administration in this study population, inclusion of dogs receiving NSAIDs was permitted providing creatinine was within normal limits prior to starting NSAIDs and at the start of the study period.

There are challenges associated with the retrospective identification of decreased kidney function and the utility of currently available grading and staging systems for AKI, acute kidney disease (AKD) and CKD [36, 37]. The kidney's response to a toxic insult can be highly variable, ranging from complete recovery to the development of fulminant kidney failure, with outcome typically only determined through careful monitoring. However, it is widely accepted that there is a relationship between serial AKI events, the incited pathophysiological response, and the future risk of CKD development defined as alteration in kidney structure or function for > 90 days [38]. Identification of AKI secondary to drug administration requires serial assessment of markers that are sensitive to either change in GFR or that indicate direct kidney injury (e.g., markers of direct tubular insult). In the current study, two definitions were used to evaluate alterations in kidney function secondary to carboplatin administration.

Decreased kidney function was defined as a sustained increase in creatinine of ≥ 26.5 μmol/L on ≥ 2 consecutive measurements. Based on IRIS AKI guidelines [28], the definition was adapted to recognise the inconsistent intervals between creatinine measurements in the study population and minimise the impact of transient, non‐pathologic fluctuations, such as those caused by hydration status. Although a creatinine increase of ≥ 26.5 μmol/L is small, due to the inverse and exponential relationship between creatinine and GFR, small increases in creatinine, particularly when persistent, can reflect a larger and clinically relevant reduction in GFR, especially in non‐azotaemic patients [39]. This is particularly pertinent when it is recognised that serum creatinine variation in an individual dog is minimal even over many years [40]. This method provided greater specificity that a decrease in kidney function had occurred.

The number of ‘creatinine, high’ AEs according to the VCOG‐CTCAE v2 (Table 1) were also determined and graded for dogs in each group [27]. The VCOG‐CTCAE system represents a highly sensitive grading scheme for the detection of change in kidney function and should be used cautiously for monitoring potential renal AEs during chemotherapy, particularly where there has been no standardisation of inter‐interval creatinine assessment. Users may choose to either grade AEs based on creatinine concentration compared to baseline, or creatinine compared to the upper limit of normal (ULN). If the former is elected, then any creatinine concentration above baseline is classified as a grade one AE. This will likely classify many dogs with slight biological or analytical variation in creatinine concentration as having experienced a chemotherapy‐related AE. Equally, however, if users were to elect to grade based on the ‘ULN’, then potentially clinically significant changes in creatinine within the reference interval may be overlooked.

In the carboplatin group, the incidence of decreased kidney function across the study period was 8.2% (*n* = 4), compared to 4.1% (*n* = 2) in the control group, which was not significantly different. Analysis did not identify any other risk factors that could explain the incidence of decreased kidney function, although, given the small number of affected dogs, it was potentially underpowered to do so and was only assessed in the carboplatin group. The incidence of decreased kidney function across both groups (6/98 dogs [6.1%]) may be explained by a proportion of the study population being exposed to other potential AKI events such as iohexol exposure, sedation and GA, and NSAID administration; a small proportion of dogs also had kidney changes, such as infarcts, present at the start of the study period, consistent with IRIS stage 1 CKD. These risk factors were not significantly different between groups, so they are less likely to contribute to the numerical difference in incidence of decreased kidney function between groups, but they may contribute to increased susceptibility to injury and the overall incidence across the study population. There is limited literature reporting the incidence of nephrotoxicity in dogs following exposure to risk factors such as GA and NSAID administration, although one study reported contrast‐induced kidney injury with 7.6% of iodinated contrast agent administrations [41]. In people, the risk of NSAID‐induced AKI varies depending on the patient population but is approximately 3% [35]. It is important to note that only three (50%) of the dogs that demonstrated decreased kidney function in the present study had creatinine increases above the reference interval, so relying on the development of overt azotaemia to identify dogs with potential decreasing kidney function is not advisable.

Using the VCOG‐CTCAE system of ‘creatinine higher than baseline’ a high proportion of dogs, 69.4% and 73.5% in the carboplatin and control groups respectively, had at least one creatinine measurement classed as a grade one or higher AE. However, there was no significant difference in the frequency of grade one, two or three creatinine AEs between groups further supporting carboplatin as a drug with limited nephrotoxic potential. Given limitations of the VCOG‐CTCAE system for accurate representation of either AKI or sustained kidney injury, it may be preferable to monitor and grade renal AEs using the VCOG and/or IRIS AKI or CKD definitions; alignment of the VCOG‐CTCAE definitions with updated AKI grading systems should also be considered.

In addition to analysing dogs on an individual level, the study also compared creatinine trends between groups with the goal of identifying more subtle differences over time. The median change in creatinine over the study period was not significantly different between groups and was only +2.0 μmol/L. This should be interpreted in the context of the significant difference in median duration of study period between the carboplatin group (138 days) and the control group (197 days). However, creatinine trends of individual dogs in each group were also analysed with a linear mixed effects model, and creatinine regression gradients did not significantly differ between groups. This difference in study duration between groups may have been influenced by the difference in distribution of cancer types between groups, and their different management approaches and prognoses. The shorter study period duration in the carboplatin group may have led to under‐reporting of decreased kidney function relative to the control group.

Whilst small changes in creatinine concentration can highlight clinically significant reductions in GFR, other surrogate markers of GFR now exist such as symmetric dimethylarginine (SDMA) which, when persistently elevated, may detect GFR changes earlier than serum creatinine; however, no dogs in the present study had SDMA data available [42]. In people, carboplatin‐associated decreased kidney function mostly manifests as subclinical reductions in GFR, which can occur within the first few weeks of treatment. GFR data was not available in the study population as, unlike in cats, it is not currently routinely measured in dogs receiving carboplatin [13, 43, 44]. Hypomagnesaemia is also commonly observed in people receiving carboplatin, with magnesium supplementation in these patients potentially providing a nephroprotective effect [45]. Serum magnesium data was not available in this study as magnesium is not routinely measured in dogs, but this could be an interesting avenue for further research.

There are several limitations to this study. The study population was relatively small, limiting statistical power and increasing the risk of a type II error. Post hoc power analysis demonstrated that a total of 1076 dogs (538 per group) would be required to demonstrate a statistically significant difference in risk of decreased kidney function between groups at the incidence rates reported in this study. Replicating our study on that scale would represent a significant challenge, but this calculation highlights that, although not statistically significant, the increased incidence of decreased kidney function in the carboplatin group may be clinically relevant and requires further attention. Case selection bias is an additional limiting factor. Several dogs were excluded from the study as they only had one creatinine measurement following baseline assessment; these dogs may have experienced increased creatinine, but were not included in the study.

The definition of decreased kidney function in the present study attempted to reduce the impact of biological and analytical variability in creatinine concentration by documenting persistently increased creatinine concentration. However, it is recognised that some dogs may have been falsely classified as having experienced carboplatin‐associated decreased kidney function, as it cannot be excluded that events occurring within inter‐measurement periods might have contributed to documented changes in creatinine concentration. Equally, episodes of carboplatin‐associated AKI and AKD could have been missed. AKI and AKD can be recognised not only by documenting decreasing GFR but may also be identified, potentially at an earlier timepoint, using urinary biomarkers that indicate direct and active tubular injury such as neutrophil gelatinase‐associated lipocalin, N‐acetyl‐glucuronidase, gamma glutamyl transferase, and cystatin B; however, these were not assessed in the current study [46, 47, 48]. For full evaluation of the impact of carboplatin administration on the kidney, prospective studies that explore such biomarkers in the appropriate time frame of AKI (i.e., 7 days) and AKD (90 days), as well as evaluation of change in GFR either through surrogate markers (creatinine, SDMA) or direct assessment (e.g., iohexol clearance, exogenous/endogenous creatinine clearance), together with longer‐term monitoring for the future development of CKD, would be required but were beyond the scope of this study.

Other limitations include the fact that data confirming a fasted status was not available from the medical records, although it is routine for owners to be asked to fast their dogs prior to appointments. Dietary protein intake is associated with serum creatinine in people; however, recent literature suggests this has minimal influence on creatinine concentration in dogs [49, 50]. For study inclusion, dogs were required to have a baseline creatinine concentration within the reference interval. Although this was intended to reduce possible confounding effects of primary kidney diseases, it may have lowered the incidence of decreased kidney function and means the results cannot be applied to azotaemic dogs receiving carboplatin. Due to the paucity of data within the clinical records, the study was also unable to identify the cause of decreased kidney function where it was identified; dogs frequently did not have urinalysis, blood pressure, or other renal biomarkers analysed alongside creatinine. Recording of serial body weights throughout the study period and comparison between groups was also not performed; severe weight loss or cachexia could result in lower creatinine concentrations due to muscle loss. It should be noted that the creatinine monitoring interval was not standardised and was longer (median 105 days) in the control group compared to the carboplatin group (median 42 days), potentially reducing the likelihood of documenting a sustained creatinine increase in the control population. Additionally, not all dogs in the carboplatin group had a creatinine measurement following completion of the chemotherapy course. Without longer‐term follow‐up, it is not possible to know whether there was progression or resolution of documented decreased kidney function. Finally, given the limited follow‐up period in this study, conclusions only relate to the short‐term effects of carboplatin administration and cannot be assumed to reflect longer‐term outcomes.

In conclusion, this study did not find a significantly increased risk of short‐term decreased kidney function in dogs receiving carboplatin compared to cancer‐bearing dogs undergoing monitoring only, although the increased incidence in the carboplatin group may be clinically relevant. Further research should aim to explore this further in larger cohorts with longer follow‐up, prospectively investigate the role of more sensitive or kidney injury‐specific biomarkers, and quantify any subclinical changes in GFR that may occur secondary to carboplatin in dogs. Additionally, the impact of carboplatin on kidney function should also be investigated in an azotaemic population.

## Conflicts of Interest

The authors declare no conflicts of interest.

## Supporting information


**Supplementary Table 1:** Frequency of dog breeds in both carboplatin (*n* = 49) and control (*n* = 49) groups.
**Supplementary Table 2:** Frequency of cancer types in both carboplatin (*n* = 49) and control (*n* = 49) groups.

## Data Availability

The data that support the findings of this study are available from the corresponding author upon reasonable request.

## References

[1] E. V. Sazonova , G. S. Kopeina , E. N. Imyanitov , and B. Zhivotovsky , “Platinum Drugs and Taxanes: Can We Overcome Resistance?,” Cell Death Discovery 7, no. 1 (2021): 155, 10.1038/s41420-021-00554-5. 34226520 PMC8257727

[2] E. Reed , “Platinum‐DNA Adduct, Nucleotide Excision Repair and Platinum Based Anti‐Cancer Chemotherapy,” Cancer Treatment Reviews 24, no. 5 (1998): 331–344, 10.1016/s0305-7372(98)90056-1. 9861196

[3] M. D. Hall , M. Okabe , D. W. Shen , X. J. Liang , and M. M. Gottesman , “The Role of Cellular Accumulation in Determining Sensitivity to Platinum‐Based Chemotherapy,” Annual Review of Pharmacology and Toxicology 48 (2008): 495–535, 10.1146/annurev.pharmtox.48.080907.180426. 17937596

[4] G. Y. Ho , N. Woodward , and J. I. Coward , “Cisplatin Versus Carboplatin: Comparative Review of Therapeutic Management in Solid Malignancies,” Critical Reviews in Oncology/Hematology 102 (2016): 37–46, 10.1016/j.critrevonc.2016.03.014. 27105947

[5] H. R. Brady , B. C. Kone , M. E. Stromski , M. L. Zeidel , G. Giebisch , and S. R. Gullans , “Mitochondrial Injury: An Early Event in Cisplatin Toxicity to Renal Proximal Tubules,” American Journal of Physiology 258, no. 5 Pt 2 (1990): F1181–F1187, 10.1152/ajprenal.1990.258.5.F1181. 2159714

[6] M. S. Park , M. De Leon , and P. Devarajan , “Cisplatin Induces Apoptosis in LLC‐PK1 Cells via Activation of Mitochondrial Pathways,” J Am Soc Nephrol 13, no. 4 (2002): 858–865, 10.1681/ASN.V134858. 11912244

[7] S. M. Somani , K. Husain , C. Whitworth , G. L. Trammell , M. Malafa , and L. P. Rybak , “Dose‐Dependent Protection by Lipoic Acid Against Cisplatin‐Induced Nephrotoxicity in Rats: Antioxidant Defense System,” Pharmacology & Toxicology 86, no. 5 (2000): 234–241, 10.1034/j.1600-0773.2000.d01-41.x. 10862506

[8] K. Tsuruya , T. Ninomiya , M. Tokumoto , et al., “Direct Involvement of the Receptor‐Mediated Apoptotic Pathways in Cisplatin‐Induced Renal Tubular Cell Death,” Kidney International 63, no. 1 (2003): 72–82, 10.1046/j.1523-1755.2003.00709.x. 12472770

[9] R. M. Franke , A. M. Kosloske , C. S. Lancaster , et al., “Influence of Oct1/Oct2‐Deficiency on Cisplatin‐Induced Changes in Urinary N‐Acetyl‐Beta‐D‐Glucosaminidase,” Clinical Cancer Research 16, no. 16 (2010): 4198–4206, 10.1158/1078-0432.CCR-10-0949. 20601443 PMC3514415

[10] R. Safirstein , P. Miller , and J. B. Guttenplan , “Uptake and Metabolism of Cisplatin by Rat Kidney,” Kidney International 25, no. 5 (1984): 753–758, 10.1038/ki.1984.86. 6540826

[11] S. Harrach and G. Ciarimboli , “Role of Transporters in the Distribution of Platinum‐Based Drugs,” Frontiers in Pharmacology 6 (2015): 85, 10.3389/fphar.2015.00085. 25964760 PMC4408848

[12] X. Yao , K. Panichpisal , N. Kurtzman , and K. Nugent , “Cisplatin Nephrotoxicity: A Review,” American Journal of the Medical Sciences 334, no. 2 (2007): 115–124, 10.1097/MAJ.0b013e31812dfe1e. 17700201

[13] T. L. Cornelison and E. Reed , “Nephrotoxicity and Hydration Management for Cisplatin, Carboplatin, and Ormaplatin,” Gynecologic Oncology 50, no. 2 (1993): 147–158, 10.1006/gyno.1993.1184. 8375728

[14] B. J. Foster , K. Clagett‐Carr , B. Leyland‐Jones , and D. Hoth , “Results of NCI‐Sponsored Phase I Trials With Carboplatin,” Cancer Treatment Reviews 12, no. Suppl A (1985): 43–49, 10.1016/0305-7372(85)90017-9. 3910221

[15] R. Skinner , A. Parry , L. Price , M. Cole , A. W. Craft , and A. D. Pearson , “Persistent Nephrotoxicity During 10‐Year Follow‐Up After Cisplatin or Carboplatin Treatment in Childhood: Relevance of Age and Dose as Risk Factors,” European Journal of Cancer 45, no. 18 (2009): 3213–3219, 10.1016/j.ejca.2009.06.032. 19850470

[16] D. T. Sleijfer , E. F. Smit , S. Meijer , N. H. Mulder , and P. E. Postmus , “Acute and Cumulative Effects of Carboplatin on Renal Function,” British Journal of Cancer 60, no. 1 (1989): 116–120, 10.1038/bjc.1989.233. 2679841 PMC2247337

[17] P. Lelieveld , W. J. van der Vijgh , and D. van Velzen , “Preclinical Toxicology of Platinum Analogues in Dogs,” European Journal of Cancer & Clinical Oncology 23, no. 8 (1987): 1147–1154, 10.1016/0277-5379(87)90148-9. 3308482

[18] R. L. Page , M. C. McEntee , S. L. George , et al., “Pharmacokinetic and Phase I Evaluation of Carboplatin in Dogs,” Journal of Veterinary Internal Medicine 7, no. 4 (1993): 235–240, 10.1111/j.1939-1676.1993.tb01013.x. 8246213

[19] M. L. Musser , K. M. Curran , B. K. Flesner , and C. M. Johannes , “A Retrospective Evaluation of Chemotherapy Overdoses in Dogs and Cats,” Frontiers in Veterinary Science 8 (2021): 718967, 10.3389/fvets.2021.718967. PMC8492923 34631850

[20] M. Iwano , K. Sadahiro , T. Maruo , S. Kawarai , H. Kayanuma , and K. Orito , “Serum Concentration and Safety of Intravenous Drip Versus Subcutaneous Administration of Carboplatin in Dogs,” Journal of Veterinary Medical Science 83, no. 5 (2021): 775–779, 10.1292/jvms.20-0653. PMC8182319 33716231

[21] G. Dank , K. M. Rassnick , Y. Sokolovsky , et al., “Use of Adjuvant Carboplatin for Treatment of Dogs With Oral Malignant Melanoma Following Surgical Excision,” Veterinary and Comparative Oncology 12, no. 1 (2014): 78–84, 10.1111/j.1476-5829.2012.00338.x. 22737988

[22] B. Phillips , B. E. Powers , W. S. Dernell , et al., “Use of Single‐Agent Carboplatin as Adjuvant or Neoadjuvant Therapy in Conjunction With Amputation for Appendicular Osteosarcoma in Dogs,” Journal of the American Animal Hospital Association 45, no. 1 (2009): 33–38, 10.5326/0450033. 19122062

[23] C. Pritchard , S. Al‐Nadaf , R. B. Rebhun , J. L. Willcox , K. A. Skorupski , and A. Lejeune , “Efficacy and Toxicity of Carboplatin in the Treatment of Macroscopic Mesenchymal Neoplasia in Dogs,” Veterinary and Comparative Oncology 21, no. 4 (2023): 717–725, 10.1111/vco.12936. 37705417

[24] K. M. Rassnick , D. M. Ruslander , S. M. Cotter , et al., “Use of Carboplatin for Treatment of Dogs With Malignant Melanoma: 27 Cases (1989‐2000),” Journal of the American Veterinary Medical Association 218, no. 9 (2001): 1444–1448, 10.2460/javma.2001.218.1444. 11345308

[25] A. C. Santamaria , J. O. Simcock , and C. A. Kuntz , “Adverse Events and Outcomes in Dogs With Appendicular Osteosarcoma Treated With Limb Amputation and a Single Subcutaneous Infusion of Carboplatin,” Journal of the American Veterinary Medical Association 255, no. 3 (2019): 345–351, 10.2460/javma.255.3.345. 31298641

[26] J. O. Simcock , S. S. Withers , C. Y. Prpich , C. A. Kuntz , and B. E. Rutland , “Evaluation of a Single Subcutaneous Infusion of Carboplatin as Adjuvant Chemotherapy for Dogs With Osteosarcoma: 17 Cases (2006‐2010),” Journal of the American Veterinary Medical Association 241, no. 5 (2012): 608–614, 10.2460/javma.241.5.608. 22916858

[27] A. K. LeBlanc , M. Atherton , R. T. Bentley , et al., “Veterinary Cooperative Oncology Group‐Common Terminology Criteria for Adverse Events (VCOG‐CTCAE v2) Following Investigational Therapy in Dogs and Cats,” Veterinary and Comparative Oncology 19, no. 2 (2021): 311–352, 10.1111/vco.12677. PMC8248125 33427378

[28] Society IRI , IRIS Guideline Reccomendations for Grading of AKI in Dogs and Cats, accessed October 19, 2024. http://www.iris‐kidney.com/guidelines/grading.html.

[29] C. A. London , A. L. Hannah , R. Zadovoskaya , et al., “Phase I Dose‐Escalating Study of SU11654, a Small Molecule Receptor Tyrosine Kinase Inhibitor, in Dogs With Spontaneous Malignancies,” Clinical Cancer Research 9, no. 7 (2003): 2755–2768. 12855656

[30] A. Matsuyama , J. P. Woods , and A. J. Mutsaers , “Evaluation of Toxicity of a Chronic Alternate Day Metronomic Cyclophosphamide Chemotherapy Protocol in Dogs With Naturally Occurring Cancer,” Canadian Veterinary Journal 58, no. 1 (2017): 51–55. PMC5157738 28042155

[31] N. H. Bexfield , R. Heiene , R. J. Gerritsen , et al., “Glomerular Filtration Rate Estimated by 3‐Sample Plasma Clearance of Iohexol in 118 Healthy Dogs,” Journal of Veterinary Internal Medicine 22, no. 1 (2008): 66–73, 10.1111/j.1939-1676.2007.0035.x. 18289291

[32] J. A. Hall , M. Yerramilli , E. Obare , M. Yerramilli , L. D. Melendez , and D. E. Jewell , “Relationship Between Lean Body Mass and Serum Renal Biomarkers in Healthy Dogs,” Journal of Veterinary Internal Medicine 29, no. 3 (2015): 808–814, 10.1111/jvim.12607. 25913398 PMC4895404

[33] Y. Miyagawa , N. Takemura , and H. Hirose , “Assessments of Factors That Affect Glomerular Filtration Rate and Indirect Markers of Renal Function in Dogs and Cats,” Journal of Veterinary Medical Science 72, no. 9 (2010): 1129–1136, 10.1292/jvms.09-0443. 20410678

[34] J. P. Braun , E. Cabe , A. Geffre , H. P. Lefebvre , and C. Trumel , “Comparison of Plasma Creatinine Values Measured by Different Veterinary Practices,” Veterinary Record 162, no. 7 (2008): 215–216, 10.1136/vr.162.7.215. 18281630

[35] D. Sabatino , M. Tillman , J. Pawasauskas , and T. Brothers , “Nonsteroidal Anti‐Inflammatory Drug Induced Acute Kidney Injury; A Review and Case Study,” Journal of Renal Injury Prevention 9, no. 4 (2020): e30, 10.34172/jrip.2020.30.

[36] L. S. Chawla , R. Bellomo , A. Bihorac , et al., “Acute Kidney Disease and Renal Recovery: Consensus Report of the Acute Disease Quality Initiative (ADQI) 16 Workgroup,” Nature Reviews Nephrology 13, no. 4 (2017): 241–257, 10.1038/nrneph.2017.2. 28239173

[37] G. Segev , S. Cortellini , J. D. Foster , et al., “International Renal Interest Society Best Practice Consensus Guidelines for the Diagnosis and Management of Acute Kidney Injury in Cats and Dogs,” Veterinary Journal 305 (2024): 106068, 10.1016/j.tvjl.2024.106068. 38325516

[38] L. D. Cowgill , D. J. Polzin , J. Elliott , et al., “Is Progressive Chronic Kidney Disease a Slow Acute Kidney Injury?,” Veterinary Clinics of North America. Small Animal Practice 46, no. 6 (2016): 995–1013, 10.1016/j.cvsm.2016.06.001. 27593574

[39] A. D. Rule and J. C. Lieske , “The Estimated Glomerular Filtration Rate as a Test for Chronic Kidney Disease: Problems and Solutions,” Cleveland Clinic Journal of Medicine 78, no. 3 (2011): 186–188, 10.3949/ccjm.78a.11004. 21364163 PMC3510658

[40] J. A. Hokamp and M. B. Nabity , “Renal Biomarkers in Domestic Species,” Veterinary Clinical Pathology 45, no. 1 (2016): 28–56, 10.1111/vcp.12333. 26918420

[41] J. B. Goic , A. M. Koenigshof , L. D. McGuire , A. C. Klinger , and M. W. Beal , “A Retrospective Evaluation of Contrast‐Induced Kidney Injury in Dogs (2006‐2012),” Journal of Veterinary Emergency and Critical Care (San Antonio, Tex.) 26, no. 5 (2016): 713–719, 10.1111/vec.12511. 27557489

[42] M. B. Nabity , G. E. Lees , M. M. Boggess , et al., “Symmetric Dimethylarginine Assay Validation, Stability, and Evaluation as a Marker for the Early Detection of Chronic Kidney Disease in Dogs,” Journal of Veterinary Internal Medicine 29, no. 4 (2015): 1036–1044, 10.1111/jvim.12835. PMC4895368 26079532

[43] D. B. Bailey , K. M. Rassnick , N. L. Dykes , and L. Pendyala , “Phase I Evaluation of Carboplatin by Use of a Dosing Strategy Based on a Targeted Area Under the Platinum Concentration‐Versus‐Time Curve and Individual Glomerular Filtration Rate in Cats With Tumors,” American Journal of Veterinary Research 70, no. 6 (2009): 770–776, 10.2460/ajvr.70.6.770. 19496668

[44] R. Choudhary , M. K. Bundela , and K. Bharang , “Comparison of Renal Functions Evaluated by Measured Glomerular Filtration Rate in Patients Treated With Cisplatin, Carboplatin, and Oxaliplatin,” Cureus 15, no. 3 (2023): e36549, 10.7759/cureus.36549. 37095803 PMC10121481

[45] G. Gaughran , K. Qayyum , L. Smyth , and A. Davis , “Carboplatin and Hypomagnesemia: Is It Really a Problem?,” Asia‐Pacific Journal of Clinical Oncology 17, no. 6 (2021): 478–485, 10.1111/ajco.13481. 33052033

[46] H. Chen , Y. Avital , S. Peterson , et al., “Urinary Cystatin B as a Marker of Acute Kidney Injury in Cats,” Veterinary Journal 308 (2024): 106262, 10.1016/j.tvjl.2024.106262. 39486474

[47] K. Masarogullari , A. Camyar , A. Garip , et al., “Urinary GGT: A Cheaper Predictor of Acute Kidney Injury?,” European Journal of Internal Medicine 24 (2013): e66–e67, 10.1016/j.ejim.2013.08.161.

[48] K. Romejko , M. Markowska , and S. Niemczyk , “The Review of Current Knowledge on Neutrophil Gelatinase‐Associated Lipocalin (NGAL),” International Journal of Molecular Sciences 24, no. 13 (2023), 10.3390/ijms241310470. PMC10341718 37445650

[49] V. Vukovic , E. Hantikainen , A. Raftopoulou , et al., “Association of Dietary Proteins With Serum Creatinine and Estimated Glomerular Filtration Rate in a General Population Sample: The CHRIS Study,” Journal of Nephrology 36, no. 1 (2023): 103–114, 10.1007/s40620-022-01409-7. 35930180 PMC9894942

[50] K. C. Yi , J. C. Heseltine , N. D. Jeffery , A. K. Cook , and M. B. Nabity , “Effect of Withholding Food Versus Feeding on Creatinine, Symmetric Dimethylarginine, Cholesterol, Triglycerides, and Other Biochemical Analytes in 100 Healthy Dogs,” Journal of Veterinary Internal Medicine 37, no. 2 (2023): 626–634, 10.1111/jvim.16630. 36786663 PMC10061199

