# Supplementary material for: Evaluation of Decreased Kidney Function in Dogs Receiving Carboplatin: A Retrospective Cohort Study of 98 Dogs (2006–2024)
Source: Vet Comp Oncol. 2025 Jun 5;23(3):442–53. doi: 10.1111/vco.13069 (PMC12378092; doi:10.1111/vco.13069)
Supplement: Supplementary file 1 — Supplementary Table 1: Frequency of dog breeds in both carboplatin (n = 49) and control (n = 49) groups. Supplementary Table 2: Frequency of cancer types in both carboplatin (n = 49) and control (n = 49) groups. [file VCO-23-442-s001.docx]

| **Carboplatin (number, %)** | **Breed** | **Control (number, %)** |
| --- | --- | --- |
| 12 (24.5%) | Crossbreed | 9 (18.4%) |
| 4 (8.2%) | Cocker spaniel | 9 (18.4%) |
| 5 (10.2%) | Labrador retriever | 5 (10.2%) |
| 1 (2.0%) | Golden retriever | 3 (6.1%) |
| - | German shepherd | 3 (6.1%) |
| 2 (4.1%) | Border collie | 2 (4.1%) |
| 2 (4.1%) | American cocker spaniel | - |
| 2 (4.1%) | Border terrier | - |
| 2 (4.1%) | Giant schnauzer | - |
| 2 (4.1%) | Greyhound | - |
| 1 (2.0%) | Beagle | - |
| 1 (2.0%) | Boston terrier | - |
| 1 (2.0%) | Boxer | 2 (4.1%) |
| 1 (2.0%) | Doberman pinscher | - |
| 1 (2.0%) | Dogue de Bordeaux | - |
| 1 (2.0%) | English springer spaniel | 1 (2.0%) |
| 1 (2.0%) | English toy terrier | - |
| 1 (2.0%) | Fox terrier | - |
| 1 (2.0%) | Jack Russell terrier | 2 (4.1%) |
| 1 (2.0%) | Japanese Akita | 1 (2.0%) |
| 1 (2.0%) | Mastiff | - |
| 1 (2.0%) | Old English sheepdog | - |
| 1 (2.0%) | Rottweiler | - |
| 1 (2.0%) | Rough collie | - |
| 1 (2.0%) | Shih tzu | 2 (4.1%) |
| 1 (2.0%) | Staffordshire bull terrier | 1 (2.0%) |
| - | Cavalier King Charles spaniel | 2 (4.1%) |
| 1 (2.0%) | West Highland white terrier | - |
| - | Saluki | 1 (2.0%) |
| - | Maltese | 1 (2.0%) |
| - | Siberian husky | 1 (2.0%) |
| - | Dachshund | 1 (2.0%) |
| - | Patterdale terrier | 1 (2.0%) |
| - | Bichon Frise | 1 (2.0%) |
| - | Cairn terrier | 1 (2.0%) |

**Supplementary Table 1:** Frequency of dog breeds in both carboplatin (n = 49) and control (n = 49) groups.

| **Carboplatin (number, %)** | **Cancer Type** | **Control (number, %)** |
| --- | --- | --- |
| 9 (18.4%) | Apocrine gland anal sac adenocarcinoma | 20 (40.8%) |
| 21 (42.9%) | Osteosarcoma (appendicular) | - |
| 4 (8.2%) | Pulmonary adenocarcinoma | 8 (16.3%) |
| - | Hepatocellular carcinoma | 5 (10.2%) |
| - | Thymic epithelial tumour | 4 (8.2%) |
| 2 (4.1%) | Urothelial cell carcinoma (bladder) | - |
| 2 (4.1%) | Mesothelioma | - |
| 2 (4.1%) | Prostatic carcinoma | - |
| - | Chemodectoma | 2 (4.1%) |
| 1 (2.0%) | Malignant melanoma (oral) | 1 (2.0%) |
| 1 (2.0%) | Mammary carcinoma | 1 (2.0%) |
| 1 (2.0%) | Chondrosarcoma | - |
| 1 (2.0%) | Thyroid carcinoma | - |
| 1 (2.0%) | Gastric adenocarcinoma | - |
| 1 (2.0%) | Splenic stromal sarcoma | - |
| 1 (2.0%) | Intestinal carcinoma | - |
| 1 (2.0%) | Carcinoma of unknown primary | - |
| 1 (2.0%) | Squamous cell carcinoma (lingual) | - |
| - | Squamous cell carcinoma (nasal planum) | 1 (2.0%) |
| - | Fibrosarcoma (oral) | 1 (2.0%) |
| - | Phaeochromocytoma | 1 (2.0%) |
| - | Perianal hepatoid gland carcinoma | 1 (2.0%) |
| - | Mast cell tumour (cutaneous) | 1 (2.0%) |
| - | Poorly differentiated sarcoma (forelimb) | 1 (2.0%) |
| - | Histiocytic sarcoma (pulmonary) | 1 (2.0%) |
| - | Soft tissue sarcoma | 1 (2.0%) |

**Supplementary Table 2:** Frequency of cancer types in both carboplatin (n = 49) and control (n = 49) groups.
